# Chest compressions before defibrillation for out-of-hospital cardiac arrest: A meta-analysis of randomized controlled clinical trials

**DOI:** 10.1186/1741-7015-8-52

**Published:** 2010-09-09

**Authors:** Pascal Meier, Paul Baker, Daniel Jost, Ian Jacobs, Bettina Henzi, Guido Knapp, Comilla Sasson

**Affiliations:** 1 University of Michigan Medical Center, Cardiovascular Medicine, Ann Arbor, Michigan, USA; 2 VA Ann Arbor Healthcare System, Ann Arbor, Michigan, USA; 3 SA Ambulance Service, Eastwood, South Australia, Australia; 4 Service Medical d'Urgence, Brigade de Sapeurs-Pompiers Paris, Paris, France; 5 Discipline of Emergency Medicine, University of Western Australia, Crawley, Australia; 6 Department of Clinical Research, University of Bern Medical School, Bern, Switzerland; 7 Department of Statistics, TU Dortmund University, Germany; 8 Department of Emergency Medicine, University of Michigan Medical Center, Ann Arbor, Michigan, USA

## Abstract

**Background:**

Current 2005 guidelines for advanced cardiac life support strongly recommend immediate defibrillation for out-of-hospital cardiac arrest. However, findings from experimental and clinical studies have indicated a potential advantage of pretreatment with chest compression-only cardiopulmonary resuscitation (CPR) prior to defibrillation in improving outcomes. The aim of this meta-analysis is to evaluate the beneficial effect of chest compression-first versus defibrillation-first on survival in patients with out-of-hospital cardiac arrest.

**Methods:**

Main outcome measures were survival to hospital discharge (primary endpoint), return of spontaneous circulation (ROSC), neurologic outcome and long-term survival.

Randomized, controlled clinical trials that were published between January 1, 1950, and June 19, 2010, were identified by a computerized search using SCOPUS, MEDLINE, BIOS, EMBASE, the Cochrane Central Register of Controlled Trials, International Pharmaceutical Abstracts database, and Web of Science and supplemented by conference proceedings. Random effects models were used to calculate pooled odds ratios (ORs). A subgroup analysis was conducted to explore the effects of response interval greater than 5 min on outcomes.

**Results:**

A total of four trials enrolling 1503 subjects were integrated into this analysis. No difference was found between chest compression-first versus defibrillation-first in the rate of return of spontaneous circulation (OR 1.01 [0.82-1.26]; *P *= 0.979), survival to hospital discharge (OR 1.10 [0.70-1.70]; *P *= 0.686) or favorable neurologic outcomes (OR 1.02 [0.31-3.38]; *P *= 0.979). For 1-year survival, however, the OR point estimates favored chest compression first (OR 1.38 [0.95-2.02]; *P *= 0.092) but the 95% CI crossed 1.0, suggesting insufficient estimate precision. Similarly, for cases with prolonged response times (> 5 min) point estimates pointed toward superiority of chest compression first (OR 1.45 [0.66-3.20]; *P *= 0.353), but the 95% CI again crossed 1.0.

**Conclusions:**

Current evidence does not support the notion that chest compression first prior to defibrillation improves the outcome of patients in out-of-hospital cardiac arrest. It appears that both treatments are equivalent. However, subgroup analyses indicate that chest compression first may be beneficial for cardiac arrests with a prolonged response time.

## Background

There are an estimated 294,851 emergency medical services (EMS)-assessed out-of-hospital cardiac arrests (OHCA) in the United States each year [1,2]. The most common underlying arrhythmias of witnessed arrests are ventricular tachycardia and ventricular fibrillation [3]. Despite major attempts to improve the chain of survival, survival rates for OHCA remain the same at 7.6% for over 30 years [4]. Average rates of survival to hospital discharge are as low as 0.3% in some communities [5,6] and depend strongly not only on the time to initiation of chest compressions but also on the time until defibrillation and the underlying rhythm [3]. While the first two factors can be influenced, they cannot be performed simultaneously. Controversy about priority has resulted from experimental and clinical data.

Current guidelines of the European Resuscitation Council (ERC) and the American Heart Association (AHA) were last updated in 2005 and emphasize the importance of early defibrillation. The International Liaison Committee on Resuscitation (ILCOR), ERC and AHA clearly prioritize early defibrillation [7,8]. However, the AHA guidelines state that in cases of nonwitnessed events, one cycle of cardiopulmonary resuscitation (CPR)/chest compressions may be considered before defibrillation (class IIb recommendation) [7]. The interval from compression to defibrillation is highly critical as impaired myocardial oxygenation distinctively decreases defibrillation success rates while myocardial preoxygenation may improve outcome [9,10].

There is, however, clinical equipoise whether professional chest compression only promptly followed by defibrillation could increase myocardial "readiness" for defibrillation. Data from the first randomized clinical trials (RCT) have shown conflicting results, but most studies were limited in size and underpowered to allow definite conclusions. A recent large-scale observational study indicated potential benefit for preshock chest compressions [11].

This is the first meta-analysis to systematically review the current research on chest compression first as compared to defibrillation first on outcomes in patients with OHCA.

## Methods

The study was performed according to PRISMA guidelines (Additional file 1) [12]. Planning and study design were done by two authors (CS, PM), including creation of an electronic database with variables of interest (Microsoft Excel). Primary and secondary endpoints, variables of interest and search strategy (databases, sources for unpublished data) were defined in a strategy outline which can be obtained from study authors on request.

### Data Sources and Searches

A search was conducted of SCOPUS, MEDLINE (via PubMed), BIOS, EMBASE, the Cochrane Central Register of Controlled Trials, International Pharmaceutical Abstracts database, and Web of Science from January 1, 1950, to June 19, 2010, supplemented by the conference proceedings of the American Heart Association (2006-2009), the American College of Cardiology (2006-2010), the European Society of Cardiology (2001-2009), the symposium on Transcatheter Cardiovascular Therapeutics (2006-2009), the World Congress of Cardiology (2006-2009) and the European Resuscitation Council Scientific Symposium (2006-2009). We also considered published review articles, editorials, and Internet-based sources of information (http://www.tctmd.com, http://www.theheart.org, http://www.europcronline.com, http://www.cardiosource.com, http://www.crtonline.com and Google scholar). For details on search strategy for MEDLINE, see Additional file 2. Similar but adapted search terms were used for the other literature databases.

### Study selection

**Figure 1:**
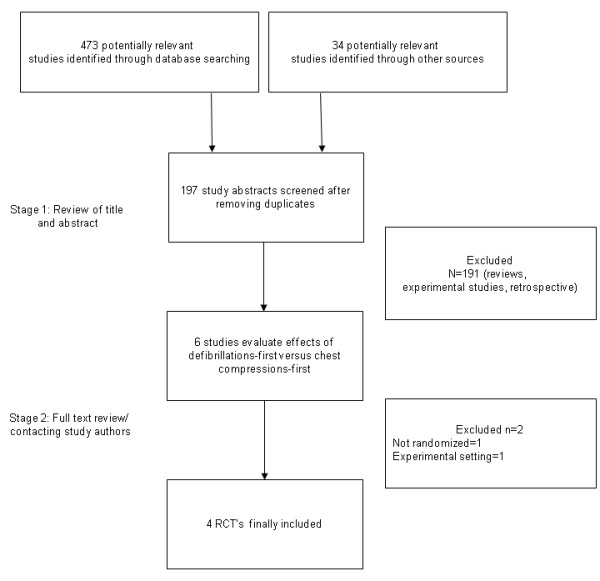
**Flow chart depicting the outline of the search and selection strategy**. RCT, randomized controlled trial.

In a two-step selection process, two investigators (PM, BH) independently reviewed the titles and abstracts of all citations to identify potentially relevant studies and to exclude duplicates. The corresponding publications were reviewed in full text by three investigators (CS, PM, BH) to assess whether studies met the following inclusion criteria: 1) randomized treatment assignment to chest compression first versus defibrillation first, 2) human study and 3) included outcome data on one of the four following clinical outcomes: return of spontaneous circulation, survival to hospital discharge, neurological outcome at discharge or survival at 1 year (Figure 1). Reviewers were not blinded to study authors or outcomes. Final inclusion of studies was based on the agreement of three investigators (CS, PM, BH).

### Data extraction and quality assessment

Relevant information from the articles, including baseline clinical characteristics of the study population and outcome measures, were extracted by two reviewers (PM, BH) using the prepared standardized extraction database (MS Excel); data on outcome (see endpoint definition below), total patient numbers per group, and covariables of interest (average age, gender, witnessed arrest, bystander CPR, response time upon arrival of emergency medical service EMS as defined by each study) were extracted. The quality of each trial was assessed using the Jadad scale to ensure sufficient quality but was not implemented in the analysis due to relevant limitations of such approaches [13,14]. Absolute numbers were recalculated when percentages were reported. All corresponding authors of included trials were contacted to ensure accuracy of the data extraction and in an attempt to obtain more information and individual patient level data.

### Endpoints

The primary endpoint of this analysis was survival to hospital discharge. However, the endpoints are presented in a chronologic order as follows:

1. Return of spontaneous circulation (ROSC)

2. Survival to hospital discharge

3. Favorable neurologic outcome at discharge (cerebral performance category (CPC) score 1 or 2)

4. Long-term outcome (survival at 1 year)

"Favorable neurological outcome" was defined as a CPC score of 1 or 2 (no or moderate cerebral disability).

### Definition of a "clinically relevant" change for the primary endpoint

We regarded a relative change of at least 20-25% as clinically relevant. Power analyses of prospective randomized trials evaluating interventions for OHCA (predefibrillation chest compression, therapeutic hypothermia) used variable definitions for "clinically relevant" differences in survival, ranging from 32-550% [15-19]. Therapeutic hypothermia as one of few measures with proven benefits in OHCA showed a 35% increase in survival in a recent meta-analysis of randomized trials [20]. Since survival is such an essential endpoint, we regard a relative change of at least 20-25% as already clinically relevant, while on the other hand, a lower threshold would not be very meaningful in the context of the general low survival to discharge rate for OHCA (average 7.6%) [4]. This would increase the risk to detect incidental differences.

### Data synthesis and analysis

All analyses were performed on an intent-to-treat basis. Data of included studies were combined to estimate the pooled treatment effect (odds ratio, OR) for the chest compression-first compared to the defibrillation-first groups. Calculations were based on a DerSirmonian and Laird random effects model [21]. Sensitivity analyses were conducted using alternative meta-analytical approaches such as the Hartung-Knapp method, which tends to be more conservative, and by meta-regression analyses (mixed-effects model) for the subgroups as defined below (R package "metafor") [22,23]. Continuity correction was used when no event occurred in one group to allow calculation of an odds ratio [24]. We used the rank correlation test to assess the risk for publication bias [25,26]. Heterogeneity among trials was quantified with Higgins's and Thompson's *I^2^*. *I*^2 ^can be interpreted as the percentage of variability due to heterogeneity between studies rather than sampling error. On the basis of findings in a previous observational study, an *a priori *subgroup analysis of response time from event to EMS arrival (≤5 min versus >5 min) was also conducted [27]. Further, a meta-regression analysis was performed on the basis of the mean response intervals of each study using a mixed-effects model. Weighted average incidence of events for the chest compression-first and the defibrillation-first groups were calculated on the basis of a random effect analysis using a Freeman-Tukey double arcsine transformation and the inverse variance method [28]. Findings are presented as point estimates and 95% confidence intervals. Analyses have been performed by two investigators independently (GK, PM). All analyses were performed with R version 2.10.1 (packages "meta," "rmeta," and "metafor") [29].

## Results

### Description of included studies

A total of 245 abstracts were reviewed, and 79 of those were subsequently reviewed as full text articles; finally, four randomized trials enrolling 1503 subjects satisfied the predetermined inclusion criteria (Figure 1) [15-18]. Tables 1, 2, 3 summarize the characteristics and quality scores of the four trials.

**Table 1:** Characteristics of included studies.

Author	Year	Location	Group	Patients (n)	Age (yrs)	Male (%)	Witnessed (%)	Bystander CPR performed (%)	Response time (min)
Jost [15]	2010	France	Defi.-first	424	62	79	86	21	10:54
			Compr.-first	421	65	78	87	21	10:30
Baker [16]	2008	Australia	Defi -first	105	66*	80	79	58	08:14
			Compr.-first	97	65*	84	84	59	07:41
Jacobs [17]	2005	Australia	Defi -first	137	62	80	74	54	09:00
			Compr.-first	119	64	80	80	64	09:20
Wik [18]	2003	Norway	Defi -first	96	80*	89	94	56	11:42
			Compr.-first	104	71*	85	91	62	12:00

*Median; Compr-first: chest compressions before defibrillation; Defi.-first: immediate defibrillation before chest compressions; response time: time-to arrival of ambulance.

**Table 2:** Characteristics of included studies.

Author	Year	Group	CPR pretreatment (sec)	Compression to ventilation ratio	No. of consecutive shocks
Jost	2010	Defi -first		Cardio-pump*	3
		Compr.-first	60	Cardio-pump*	1
Baker	2008	Defi -first			3
		Compr.-first	180	15:2	3
Jacobs	2005	Defi -first			3
		Compr.-first	90	5:1	3
Wik	2003	Defi -first			3
		Compr.-first	180	5:1	3

* Trademark (manufacturer: Ambu, Denmark). Compr-first: chest compressions before defibrillation;

Defi.-first: immediate defibrillation before chest compressions; sec: seconds

**Table 3:** Quality of included studies (Jadad score).

Author	Randomized	Appropriate randomization	Double blind	Appropriate blinding (single blind)	Drop outs appropriately declared	Score
Jost	Yes	Yes	No	Yes	Yes	4/5
Baker	Yes	Yes	No	Yes	Yes	4/5
Jacobs	Yes	Yes	No	Yes	Yes	4/5
Wik	Yes	Yes	No	Yes	Yes	4/5

### Outcomes

#### Return of spontaneous circulation (ROSC)

**Figure 2:**
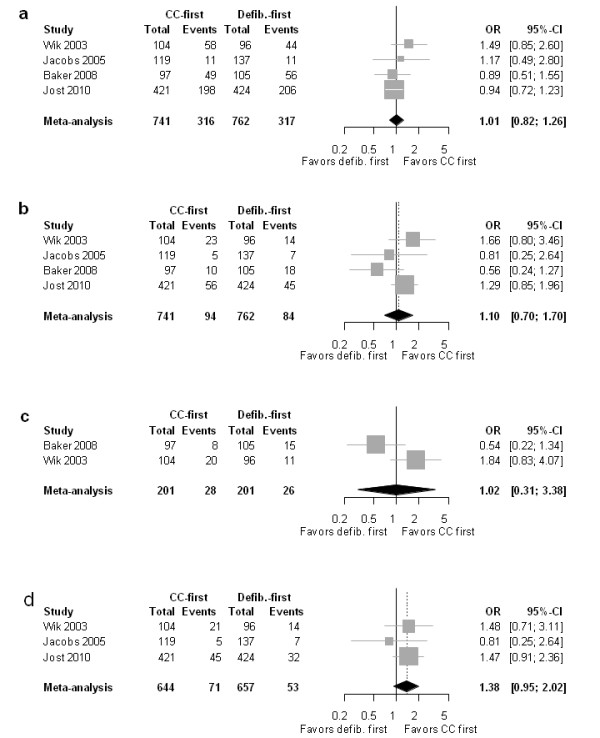
**Forest plot of odds ratios (OR) of (a) ROSC, (b) survival to hospital discharge (primary endpoint), (c) favorable neurologic outcome, and (d) 1-year survival**. Horizontal bars indicate 95% confidence intervals. Size of markers represents study weight in meta-analysis.

The pooled analysis did not reveal a relevant difference in the overall chance for ROSC between the chest compression-first and the defibrillation-first approach (OR 1.01 [0.82-1.26]; *P *= 0.979; heterogeneity: *I*^2 ^= 0%, *P *= 0.79) (Figure 2a). The weighted average proportion of patients in whom ROSC was achieved was 39.2% [19.8-60.5%] for the chest compression-first group and 37.3% [17.0-60.2%] for the defibrillation-first group.

#### Survival to hospital discharge

As summarized for all response times in Figure 2b, the direct comparison between the chest compression-first and the defibrillation-first approach did not reveal a relevant difference (OR 1.10 [0.70-1.70]; *P *= 0.686; heterogeneity: *I*^2 ^= 34.4%, *P *= 0.206). The average weighted proportion of patients able to leave the hospital after cardiac arrest was 12.0% [6.4-19.1%] for the chest compression-first group as compared to 11.4% [7.1-16.6%] for the defibrillation-first group.

#### Favorable neurologic outcome

The average weighted proportion of patients with favorable neurological status was 13.7% [4.9-25.9%] after chest compression first and 13.3% [9.0-18.3%] after defibrillation first. As seen in Figure 2c, patients who were treated with chest compression first did not show an increased likelihood of a "favorable neurologic outcome" (as defined by a CPC score of 1 or 2) compared to those with defibrillation first (OR 1.02 [0.31-3.38]; *P *= 0.979; heterogeneity: *I*^2 ^= 74.9%, *P *= 0.05).

#### One-year survival

As shown in Figure 2d, the OR point estimates favored a chest compression-first approach (OR 1.38 [0.95-2.02]; *P *= 0.092; heterogeneity: *I*^2 ^= 0%, *P *= 0.647). However, the 95% confidence intervals crossed 1.0, indicating insufficient precision of the effect size estimation and resulting in statistical nonsignificance. The average weighted proportion of patients able to leave the hospital after cardiac arrest with chest compression first it was 11.0% [4.8-19.5%] as compared to 8.6% [4.8-13.4%] for patients treated with defibrillation first.

**Figure 3:**
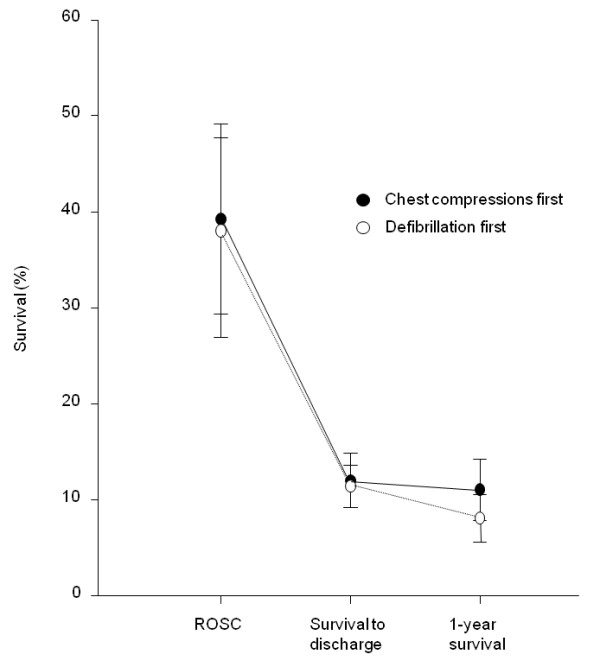
**Survival of enrolled patients after cardiac arrest (average percentage and 95% confidence intervals)**.

Figure 3 summarizes the chance of survival of patients involved in the included trials after cardiac arrest up to 1 year after the event. As mentioned above, ROSC was achieved in approximately 40% of patients with OHCA included in these trials, chance for survival to hospital discharge was around 12.0% and similar between both treatment groups, while the survival chance at 1 year was 11.0% with chest compression first and 8.6% with defibrillation first.

#### Subgroup Analyses Based on Response Intervals (Call to EMS Arrival)

**Figure 4:**
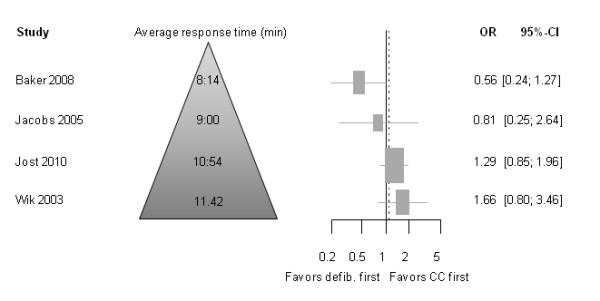
**Odds ratio (OR) for primary endpoint "survival to hospital discharge" and response time**. Horizontal bars indicate 95% confidence intervals. Size of markers represents study weight in meta-analysis.

In Figure 4, the studies are ordered according to their average EMS response times. OR point estimates of studies with shorter EMS response times favored a defibrillation-first approach. The longer the EMS response times, the OR point estimates favored chest compression first followed by defibrillation. However, for all these OR estimates, the 95% confidence intervals crossed 1.0; thus, none of the differences were statistically significant.

#### Response Interval ≤5 minutes

##### ROSC

**Figure 5:**
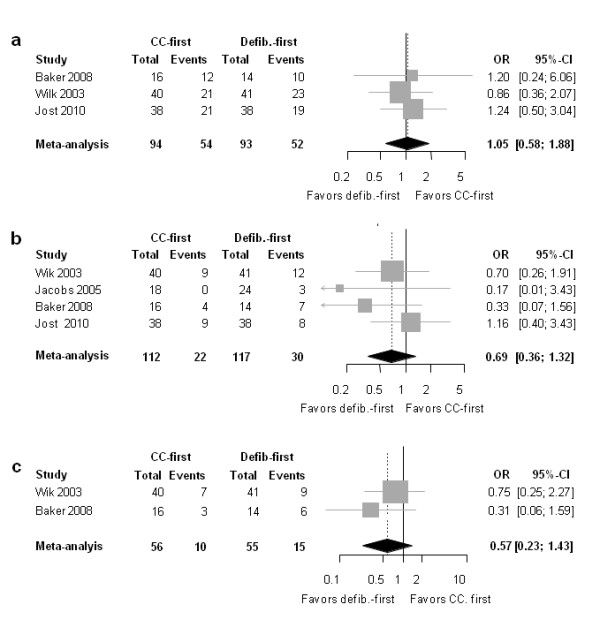
**Forest plot of odds ratios (OR) of the subgroup of patients with response time ≤5 min for (a) ROSC, (b) survival to hospital discharge, and (c) favorable neurologic outcome**. Horizontal bars indicate 95% confidence intervals. Size of markers represents study weight in meta-analysis.

As shown in Figure 5a, for response time ≤5 minutes, the OR to achieve ROSC was not significantly different between chest compression first and defibrillation first (OR 1.05 [0.58-1.88]; *P *= 0.872; heterogeneity: *I*^2 ^= 0%, *P *= 0.73).

##### Survival to discharge

The point estimates of the OR for this outcome were in disfavor of predefibrillation chest compressions (OR 0.69 [0.36-1.32]; *P *= 0.263; heterogeneity: *I*^2 ^= 0%, *P *= 0.954) (Figure 5b). The 95% confidence interval crossed 1.0, indicating inadequate precision of the effect estimate, resulting in statistical nonsignificance.

##### Neurologic outcome

As Figure 5c shows, the OR point estimate was in disfavor of predefibrillation chest compression approach (OR 0.57 [0.23-1.43]; *P *= 0.300 (heterogeneity: 0%; *P *= 0.370). Again, the 95% confidence interval crossed 1.0, and the difference was therefore not statistically significant.

#### Response Interval >5 minutes

##### ROSC

**Figure 6:**
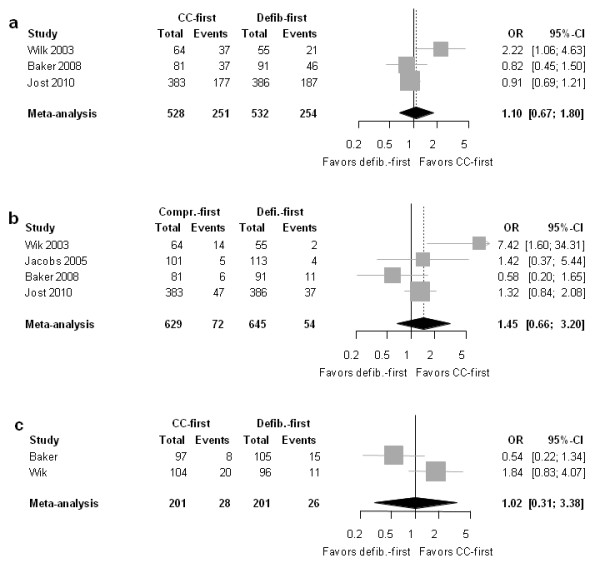
**Forest plot of odds ratios (OR) of group with response time >5 min for (a) ROSC, (b) survival to hospital discharge and (c) good neurologic outcome**. Horizontal bars indicate 95% confidence intervals. Size of markers represents study weight in meta-analysis.

No relevant differences were found for patients with a response time >5 minutes in ROSC (Figure 6a), the OR was 1.10 [0.67-1.78]; *P *= 0.705 (heterogeneity: 62.4%; *P *= 0.0712).

##### Survival to discharge

The point estimate for the OR pointed toward superiority of chest compression first, but the confidence interval crossed 1.0; thus, the finding was not statistically significant (OR 1.45 [0.66-3.20]; *P *= 0.353; heterogeneity: 59.1%; *P *= 0.062) (Figure 6b).

##### Neurologic outcome

As Figure 6c illustrates, there was no relevant difference between the two groups (OR 1.02 [0.31-3.38]; *P *= 0.879; heterogeneity: *I*^2 ^= 84.2%; *P *= 0.012).

### Meta-regression analysis based on mean response intervals

This analysis showed a significant effect of the mean response interval of each study in the control arm on the effect of predefibrillation chest compression; the point estimates of the OR pointed toward inferiority of predefibrillation chest compression for studies with short mean response intervals but toward superiority for studies with longer mean response intervals (Additional file 3; Supplementary Figure 1). This response interval effect was statistically significant. The slope of the meta-regression was 0.0051 [0.0004-0.0097]; *P *= 0.033. That is, for every absolute increase of 1 time unit (1 second) in the response time, the log odds ratio increased by 0.0051 (in direction to superiority of a chest compression-first approach). At around 600 seconds (10 min) response time, the regression line crosses OR 1.0 (equipoise between the two interventions). Additional file 4, Supplementary Table 6 gives an overview of variable response intervals with corresponding predicted odds ratios.

### Sensitivity analyses

The analysis performed with the Hartung-Knapp meta-analytical approach and by a mixed-effects meta-regression analysis revealed almost identical results (see Additional file 5, Supplementary Tables 3-5. Also, a sensitivity analysis was conducted without the study by Jost et al. [15], as this study did not exclusively test the effect of chest compression first, but also the effect of three consecutive shock applications versus a single shock at a time. Also, most patients did not receive bystander CPR; CPR was initiated in most cases by firefighters using a CPR device instead of manual compressions. When excluding this study, the results did not change despite the considerable weight (study size) of this study in this analysis (data not presented).

### Publication bias assessment

Regarding the primary endpoint, the rank correlation test was not suggestive for publication bias, *P *= 0.588.

## Discussion

This is the first meta-analysis evaluating the effect of chest compression first versus defibrillation first in patients having out-of-hospital cardiac arrest. We included four randomized, controlled clinical trials with 1503 subjects. Overall, our findings suggest that there was no significant difference between the two groups in general. However, our subgroup analyses of patients with a response interval >5 min found point estimates that pointed toward superiority of a chest compression-first approach and vice versa for the subgroup with response interval ≤5 min. The point estimate for the 1-year survival results pointed toward a lower 1-year mortality for chest compression-first patients, which was mainly driven by studies with longer EMS response times [15,18]. However, the 95% confidence intervals of these subgroup and long-term analyses crossed 1.0, indicating insufficient precision of the effect estimates and resulting in statistical nonsignificance. These analyses were based on smaller patient numbers.

### Rational for Chest Compressions Prior to Defibrillation

Chest compressions serve to empty the right ventricle (RV) and to avoid RV distension during VF, which helps to reduce the risk of occurrence of "nonperfusing" postdefibrillation rhythms (e.g., pulseless electrical activity or asystole) [30,31]. Two experimental animal studies on ventricular defibrillation have demonstrated that chest compression first may improve defibrillation success in comparison to the standard defibrillation first approach. A randomized study in swine conducted by Berg et al. and a study by Niemann et al. in dogs both showed higher efficiency for chest compression prior to defibrillation [32,33]. Data from a study conducted on humans showed that even short preshock pauses were found to strongly correlate with lower defibrillation success [34]. Accordingly, a large observational study by Cobb et al. demonstrated improved survival for patients treated for out-of-hospital cardiac arrest after implementation of chest compression-first protocol compared to the preceding 42 months with the standard defibrillation-first approach [27]. Similarly, a study including 886 patients of Bobrow et al. performed in Arizona implementing a protocol of 200 uninterrupted chest compressions before defibrillation (single shock) showed a remarkable increase in survival-to-hospital discharge, from 1.8% to 5.4% after protocol implementation [35,36]. Yet, despite all of the above data from experimental and observational studies, our meta-analysis based on randomized clinical trials in humans shows that both treatments appear to be equivocal, with point estimates that favor chest compression first regarding long-term outcomes.

Several aspects could explain this controversy. First, findings from experimental animal studies may not apply to humans, especially since most models use electrical induction of ventricular fibrillation, which may not appropriately reflect the majority of cardiac arrests in humans [37]. In a more recent study in swine using an acute myocardial ischemia model, 24-hr survival with a favorable neurological outcome was less likely when chest compressions were performed prior to defibrillation [38]. Second, observational studies [27,35] are more prone to confounding than randomized trials. Because we decided *a priori *to include only randomized, controlled trials in our meta-analysis, our results may differ from these large observational studies. Finally, it may be that the treatment effect of chest compression first may be dependent on the response interval from the time of call to EMS response. Further research, with patient-level data, will need to be conducted to assess whether this finding is consistent.

### Short- versus longer-duration cardiac arrest

The possible difference in treatment effect for longer-lasting (response interval >5 min) makes plausible sense from a pathophysiological standpoint. Cardiac arrest (due to ventricular tachycardia/fibrillation (VT/VF)) is definitively not a static event. Rather, it is a dynamic process with sometimes continuous transitions starting with VT, transforming into coarse and then into fine amplitude VF and finally into asystole; these different electrocardiogram morphologies are obviously associated with different degrees of defibrillation success [39]. During the course of VF high-energy phosphates are progressively depleted, which also decreases the chances for successful defibrillation [40].

Niemann et al. demonstrated the superiority chest compression first in a dog model [33], but found better outcomes for defibrillation first in a subsequent study [41]. In this second study, VF duration was relevantly shorter (5 min versus 7.5 min in the first study). Another study conducted in dogs specifically evaluated different VF durations, showing differential results based on the duration of VF. For short-lasting VF arrests (< 3 min), defibrillation first was superior to chest compression first [42]. It has to be considered, however, that most experimental animal studies used electrical induction of VF, which may not be identical to ischemia-induced VF [37]. The study by Cobb et al. included in our analysis showed the most prominent benefit for chest compression first if response time was >4 min [27].

In 2002, Weisfeldt et al. proposed a three-phase time-sensitive model for treatment of sudden cardiac arrest: the electrical phase (early phase during the first around 0-4 min where immediate defibrillation may be optimal, the circulatory phase (4-10 min) where predefibrillation chest compressions could be meaningful, and the metabolic phase (> 10 min), where survival rates are poor in general [39]. The authors stated in their editorial that "phase-specific research is needed to extend knowledge of the importance of time on resuscitation, such as testing early defibrillation and public access defibrillation programs during the electrical phase and testing chest compression and vasoconstrictors first during the circulatory phase." [39]. Our findings support the view of Weisfeld et al. as illustrated in Figure 4 and as shown in the subgroup analyses of patients with longer versus those with shorter response intervals.

### Limitations of this study

It has to be considered that nonstratified overall results showed odds ratios very close to 1.0; that is, no treatment effect with fairly narrow confidence (precision) intervals and with very little heterogeneity. In contrast, OR point estimates pointed toward superiority of predefibrillation chest compressions for those cardiac arrests with prolonged EMS response, while in patients with shorter EMS intervals these OR estimates pointed toward superiority of a defibrillation-first approach (Figures 5 and 6). Owing to the smaller sample sizes in these subgroups, confidence intervals were wider due to reduced precision of these estimates. The confidence intervals for these subgroup analyses crossed 1.0; i.e., the result was statistically not significant. It is possible that there is in fact a difference that was not detected by our analysis due to limited statistical power. An interaction between optimal treatment and response time is further supported by the observation that the odds ratios were influenced by the average response intervals of the individual studies (Figure 3 and Additional file 1). However, the meta-regression analysis (Additional file 1), even though in line with the findings of the subgroup analyses, has to be interpreted with care because it is based on summary measure (mean response intervals of each study) and not on individual response intervals. Meta-analyses are useful for synthesizing the literature and to explore areas for further exploration rather than to provide a definitive conclusion. Future research based on this meta-analysis could be conducted with patient-level data to assess whether the overall pooled results are consistent with the individual-level data.

RCT data are considered the "golden standard" and superior to observational studies. Clearly, the latter are more prone to be biased by confounding, and, accordingly, we considered RCT exclusively in this meta-analysis. Nevertheless, there are caveats for RCT also [43]; this is especially true in the context of human emergency medicine research. The vast majority of patients assessed for inclusion in these trials were finally not eligible because of predefined exclusion criteria or owing to logistical reasons. Thus, the patient selection associated with RCT potentially complicates generalizability of findings into routine clinical practice. For example, bystander CPR rate ranged from 54-64% in three of the included trials, while the AHA estimates the average bystander CPR rate in the United States to be 31.4% [1]. Future research will need to be conducted on communities that may be more generalizable than the study populations in this analysis.

A further limitation of this study is the heterogeneity of the study protocols. Three of the four included trials use the 2000 guidelines with a "three-shock protocol" [16-18],

while one study utilized a single shock application (as advocated in the current 2005 guidelines) in the chest compression first group [15]. All four studies did not control for the quality of chest compressions. The quality of chest compressions has a key impact on outcome and is often insufficient, even for in-hospital cardiac arrests [34] and even in some experimental studies [44]. We cannot exclude that the quality of compressions in the included studies was insufficient, and as a consequence, the studies were unable to show a benefit. Because of the differences in study protocols, we chose to use a random effects model rather than a fixed-effect model for data analysis.

Finally, we did not have the complete set of individual patient data, and our analyses are thus based on study-level data. Therefore, we could not adjust the analysis for covariables. For example, the 1-year survival data for the study by Jost et al. [15] are based on Kaplan-Meier survival estimates, which showed a survival probability of 10.6% in the intervention group and 7.6% in the control group (*P *= 0.45).

## Conclusions

The results of this meta-analysis demonstrate that survival is equivocal for the chest compression-first group as compared to the defibrillation-first group. Thus, current guidelines emphasizing early defibrillation still appear appropriate. However, the study revealed signals toward possible superiority of predefibrillation chest compressions for patients with a response interval of >5 min; the statistical power of this study was insufficient for such subgroup analyses, and none reached statistical significance. These signals suggest that the optimal treatment of cardiac arrest patients may depend on the duration of the event and the timeliness of the response. Future research will need to be conducted to assess whether this differential effect is seen in patients treated for out-of-hospital cardiac arrest. This may lead to different treatment guidelines based on the duration of the arrest and the interval of the response.

## Abbreviations

AHA: American Heart Association; CPR: Cardiopulmonary resuscitation; ERC: European Resuscitation Council; EMS: Emergency medical services; ILCOR: International Liaison Committee on Resuscitation; OHCA: Out-of-hospital cardiac arrests; OR: Odds ratio; RCT: Randomized clinical trials; ROSC: Return of spontaneous circulation.

## Competing interests

The authors declare that they have no competing interests to disclose. PM is supported by a postdoctoral fellowship grant from the Swiss National Research Foundation, Switzerland. The funding organizations had no role in the design and conduct of the study; the collection, management, analysis, and interpretation of the data; or the preparation, review, or approval of the manuscript.

## Authors' contributions

PM, CS conceptualized and designed this meta-analysis. PM, BH, CS were substantially involved in data acquisition (literature search, study selection and data abstraction). PM, GK, CS performed the analyses and were substantially involved in data interpretation. PB, DJ, IJ provided data used for the analysis and relevantly contributed to the interpretation and intellectual content of the manuscript. PM and CS drafted the manuscript. All authors revised the manuscript critically for important intellectual content. All authors approved the final version.

## Pre-publication history

The pre-publication history for this paper can be accessed here:

http://www.biomedcentral.com/1741-7015/8/52/prepub

## Supplementary Material

Additional file 1 **Supplementary table 1**. PRISMA statement checklist. Click here for file

Additional file 2 **Supplementary table 2**. Detailed literature search strategy with search terms used for Medline. Click here for file

Additional file 3 **Supplementary figure 1**. Meta-regression plot. Meta-regression plot of odds ratios (OR) versus response interval (seconds). Size of circles indicate study weights in a mixed-effects model. Click here for file

Additional file 4 **Supplementary table 6**. Predicted odds ratios for variable response intervals. Click here for file

Additional file 5 **Supplementary tables 3 - 5**. Sensitivity analyses with different meta-analytical approaches. Click here for file
