# Supplementary material for: Chest compressions before defibrillation for out-of-hospital cardiac arrest: A meta-analysis of randomized controlled clinical trials
Source: BMC Med. 2010 Sep 9;8:52. doi: 10.1186/1741-7015-8-52 (PMC2942789; doi:10.1186/1741-7015-8-52)
Supplement: Additional file 2 — Supplementary table 2. Detailed literature search strategy with search terms used for Medline. [file 1741-7015-8-52-S2.DOC]

| **Search** | **# of abstracts** |
| --- | --- |

Search History

Search Queries

#13 Search #5 AND #11 473

Limits: Humans, Meta-Analysis, Randomized Controlled Trial,

Clinical Trial, Phase I, Clinical Trial, Phase II, Clinical Trial,

Phase III, Clinical Trial, Phase IV, Controlled Clinical Trial

#12 Search #5 AND #11 6478

#11 Search #6 OR #9 OR #10 36783

#10 Search "cardiac arrest"[All Fields] 15547

#9 Search "Heart Arrest"[Mesh] 26676

#6 Search "sudden cardiac death"[All Fields] 6673

#5 Search #2 OR #3 OR #4 20257

#4 Search "defibrillation"[All Fields] 5425

#3 Search "cardiopulmonary resuscitation"[Mesh] 8266

#2 Search "Electric Countershock"[Mesh] 10946

Supplementary Table 2: Search strategy for MEDLINE (search date June 19, 2010).
