# Supplementary figures and images for: Chest compressions before defibrillation for out-of-hospital cardiac arrest: A meta-analysis of randomized controlled clinical trials
Source: BMC Med. 2010 Sep 9;8:52. doi: 10.1186/1741-7015-8-52 (PMC2942789; doi:10.1186/1741-7015-8-52)

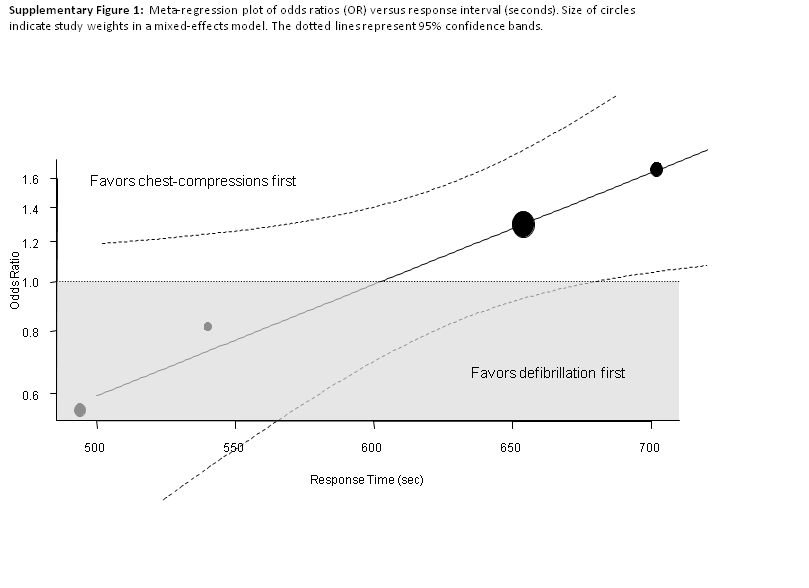

Supplement: Additional file 3 — Supplementary figure 1. Meta-regression plot. Meta-regression plot of odds ratios (OR) versus response interval (seconds). Size of circles indicate study weights in a mixed-effects model. [file 1741-7015-8-52-S3.TIFF]
