# Supplementary material for: Chest compressions before defibrillation for out-of-hospital cardiac arrest: A meta-analysis of randomized controlled clinical trials
Source: BMC Med. 2010 Sep 9;8:52. doi: 10.1186/1741-7015-8-52 (PMC2942789; doi:10.1186/1741-7015-8-52)
Supplement: Additional file 4 — Supplementary table 6. Predicted odds ratios for variable response intervals. [file 1741-7015-8-52-S4.DOC]

Supplementary Table 6: Predicted OR for variable response intervals.

| **Response time (min)** | **Predicted odds ratio** | |
| --- | --- | --- |
|  | OR | 95% CI |
| **7** | 0.40 | [0.14 - 1.12 ] |
| **8** | 0.54 | [0.25 - 1.17] |
| **9** | 0.73 | [0.43 - 1.24] |
| **10** | 0.99 | [0.70 - 1.40] |
| **11** | 1.34 | [0.95 - 1.90] |
| **12** | 1.82 | [1.07 - 3.07] |

CI: confidence interval; OR: odds ratio.
