# Supplementary material for: Chest compressions before defibrillation for out-of-hospital cardiac arrest: A meta-analysis of randomized controlled clinical trials
Source: BMC Med. 2010 Sep 9;8:52. doi: 10.1186/1741-7015-8-52 (PMC2942789; doi:10.1186/1741-7015-8-52)
Supplement: Additional file 5 — Supplementary tables 3 - 5. Sensitivity analyses with different meta-analytical approaches. [file 1741-7015-8-52-S5.DOC]

Supplementary Table 3: Sensitivity analyses with different meta-analytical approaches.

|  | **Knapp-Hartung method** | | **Random effect model (DerSirmonian-Laird method)** | | |
| --- | --- | --- | --- | --- | --- |
|  | OR | 95% CI | OR | 95% CI |  |
| **ROSC** | 1.01 | [0.74 – 1.39] | 1.01 | [0.82 - 1.26] |  |
| **Survival to hospital discharge** | 1.10 | [0.82 - 1.26] | 1.10 | [0.70 - 1.70] |  |
| **Good neurological outcome** | 1.02 | [0.01 - 10.98] | 1.02 | [0.31 - 3.38] |  |
| **1-year survival** | 1.38 | [0.80 – 2.39] | 1.38 | [0.95 - 2.02] |  |

CI: confidence interval; OR: odds ratio; ROSC: return of spontaneous circulation.

Supplementary Table 4: Sensitivity analyses for subgroups with ≤ 5 minutes response interval based on a meta-regression analysis (mixed-effects model).

|  | **Random effect model (DerSirmonian-Laird method)** | | **Meta-regression (mixed-effects model)** | |
| --- | --- | --- | --- | --- |
|  | OR | 95% CI | OR | 95% CI |
| **ROSC** | 1.05 | [0.58 **-** 1.88] | 1.05 | [0.55 - 2.00] |
| **Survival to hospital discharge** | 0.69 | [0.36 - 1.32] | 0.65 | [0.28 - 1.48] |
| **Good neurological outcome** | 0.57 | [0.23 - 1.43] | 0.53 | [0.15 - 1.90] |

CI: confidence interval; OR: odds ratio; ROSC: return of spontaneous circulation.

Supplementary Table 5: Sensitivity analyses for subgroups > 5 minutes response interval based on a meta-regression analysis (mixed-effects model).

|  | **Random effect model (DerSirmonian-Laird method)** | | | **Meta-regression (mixed-effects model)** | | |
| --- | --- | --- | --- | --- | --- | --- |
|  | OR | 95% CI | OR | | 95% CI |  |
| **ROSC** | 1.10 | [0.67 - 1.80] | 1.06 | | [0.73 - 1.54] |  |
| **Survival to hospital discharge** | 1.45 | [0.66 - 3.20] | 1.41 | | [0.72 - 2.78] |  |
| **Good neurological outcome** | 1.02 | [0.31 - 3.38] | 1.02 | | [0.36 - 2.91] |  |

CI: confidence interval; OR: odds ratio; ROSC: return of spontaneous circulation.
